# Comparison of a deep learning-accelerated T2-weighted turbo spin echo sequence and its conventional counterpart for female pelvic MRI: reduced acquisition times and improved image quality

**DOI:** 10.1186/s13244-022-01321-5

**Published:** 2022-12-13

**Authors:** Jing Ren, Yuan Li, Fei-Shi Liu, Chong Liu, Jin-Xia Zhu, Marcel Dominik Nickel, Xiao-Ye Wang, Xin-Yu Liu, Jia Zhao, Yong-Lan He, Zheng-Yu Jin, Hua-Dan Xue

**Affiliations:** 1 Department of Radiology, Peking Union Medical College Hospital, Chinese Academy of Medical Sciences and Peking Union Medical College, Shuai Fu Yuan Road, Dongcheng Dist., Beijing, 100730 People’s Republic of China; 2 Department of Obstetrics and Gynecology, Peking Union Medical College Hospital, Chinese Academy of Medical Sciences and Peking Union Medical College, National Clinical Research Center for Obstetric and Gynecologic Diseases, Beijing, People’s Republic of China; 3 MR Collaboration, Siemens Healthineers Ltd., Beijing, People’s Republic of China; 4 MR Application Predevelopment, Siemens Healthcare GmbH, Erlangen, Germany; 5 MR Clinical Marketing, Siemens Healthineers Ltd., Beijing, People’s Republic of China

**Keywords:** Magnetic resonance imaging, Female pelvis, Deep learning, Image quality, Turbo spin echo

## Abstract

**Objectives:**

To investigate the feasibility of a deep learning-accelerated T2-weighted turbo spin echo (TSE) sequence (T2_DL_) applied to female pelvic MRI, using standard T2-weighted TSE (T2_S_) as reference.

**Methods:**

In total, 24 volunteers and 48 consecutive patients with benign uterine diseases were enrolled. Patients in the menstrual phase were excluded. T2_S_ and T2_DL_ sequences in three planes were performed for each participant. Quantitative image evaluation was conducted by calculating the signal-to-noise ratio (SNR) and contrast-to-noise ratio (CNR). Image geometric distortion was evaluated by measuring the diameters in all three directions of the uterus and lesions. Qualitative image evaluation including overall image quality, artifacts, boundary sharpness of the uterine zonal layers, and lesion conspicuity were assessed by three radiologists using a 5-point Likert scale, with 5 indicating the best quality. Comparative analyses were conducted for the two sequences.

**Results:**

T2_DL_ resulted in a 62.7% timing reduction (1:54 min for T2_DL_ and 5:06 min for T2_S_ in axial, sagittal, and coronal imaging, respectively). Compared to T2_S_, T2_DL_ had significantly higher SNR (*p* ≤ 0.001) and CNR (*p* ≤ 0.007), and without geometric distortion (*p* = 0.925–0.981). Inter-observer agreement regarding qualitative evaluation was excellent (Kendall’s W > 0.75). T2_DL_ provided superior image quality (all *p* < 0.001), boundary sharpness of the uterine zonal layers (all *p* < 0.001), lesion conspicuity (*p* = 0.002, *p* < 0.001, and *p* = 0.021), and fewer artifacts (all *p* < 0.001) in sagittal, axial, and coronal imaging.

**Conclusions:**

Compared with standard TSE, deep learning-accelerated T2-weighted TSE is feasible to reduce acquisition time of female pelvic MRI with significant improvement of image quality.

## Key points

- T2_DL_ resulted in a 62.7% reduction in acquisition time compared to T2_S_
- T2_DL_ can provide higher SNR and CNR, and without geometric distortion
- T2_DL_ is feasible for reducing the motion-induced blurring and improving image quality

## Introduction

Due to its excellent soft-tissue contrast, MRI is widely used to evaluate malignant and benign diseases of the female pelvis [1–3]. T2-weighted imaging (T2WI) is the standard sequence used in gynecological MRI and a basic female pelvic MRI protocol with at least two T2WI orthogonal planes is recommended for patients with uterine disease [1, 4, 5]. However, a disadvantage of traditional pelvic MR protocol is that it has a long acquisition time, ranging from approximately 10 to 15 min for T2w sequences, which may increase waiting time for patients and reduce throughput for medical centers [6–8]. In addition, the extensive duration of individual acquisitions renders the examination motion-sensitive, often resulting in motion artifacts or image blurring, especially in elderly patients or those with claustrophobia who are unable to remain still for long periods [9].

Multiple strategies have been employed to optimize acquisition of images in a limited timeframe while maintaining sufficient diagnostic value. Efforts to reduce motion artifacts include patient preparation (e.g., use of an intravenous antiperistaltic agent, fasting, or emptying of the bladder) and scanning techniques (e.g., fat suppression, saturation bands, or signal averaging). However, these methods do not completely eradicate artifacts and may increase the cost and duration of MR examinations [5, 10, 11]. Innovative techniques that may substantially reduce scanning time while producing motion-resistant images include partial Fourier acquisition, parallel acquisition techniques, and compressed sensing (CS). Partial Fourier imaging and parallel acquisition techniques are time-saving technologies that omit phase-encoding steps in a regular fashion, but lead to SNR loss [11, 12]. CS requires an incoherent undersampling pattern; however, the images exhibit residual blurring and an unnatural appearance due to oversimplified image content [13, 14]. Although these sequences are routinely available, their clinical use is limited because of trade-offs between saving time and loss of SNR, sharpness, or natural appearance [13, 15].

With recent advances in artificial intelligence, a deep learning (DL) reconstruction method involving an unrolled variational network allows for a reduction in acquisition time while preserving image quality and realistic textures [16]. The particular feature of unrolled variational networks in the DL algorithm beyond image denoising is given by the fact that the network constantly considers data consistency with the acquired k-space data through a parallel imaging model. This allows to reduce the noise significantly and provide images with higher SNR [17]. It is therefore possible to invest the potential improvement in shorting of the acquisition time by employing higher acceleration factors or fewer averages. Previous studies have found that the DL algorithm enables to yield satisfactory image quality and significantly save time in MRI protocols for the abdomen, knee, and prostate (DL vs. conventional sequences: 0:16 vs. 4:00 min, 4:20 vs. 8:11 min, and 1:38–3:50 vs. 4:37–10:21 min, respectively) [17–20]. However, the value of DL-accelerated reconstruction technique applied to female pelvic MRI has not been studied. We hypothesized that this DL reconstruction method would accelerate the gynecological MRI protocol while producing high-quality images. To test this hypothesis, we compared the feasibility of the DL-accelerated TSE sequence (T2_DL_) with that of a standard TSE sequence (T2_S_) for gynecological T2WI by evaluating examination time, image quality, and lesion conspicuity of benign uterine diseases.

## Methods

### Study design

**Fig. 1:**
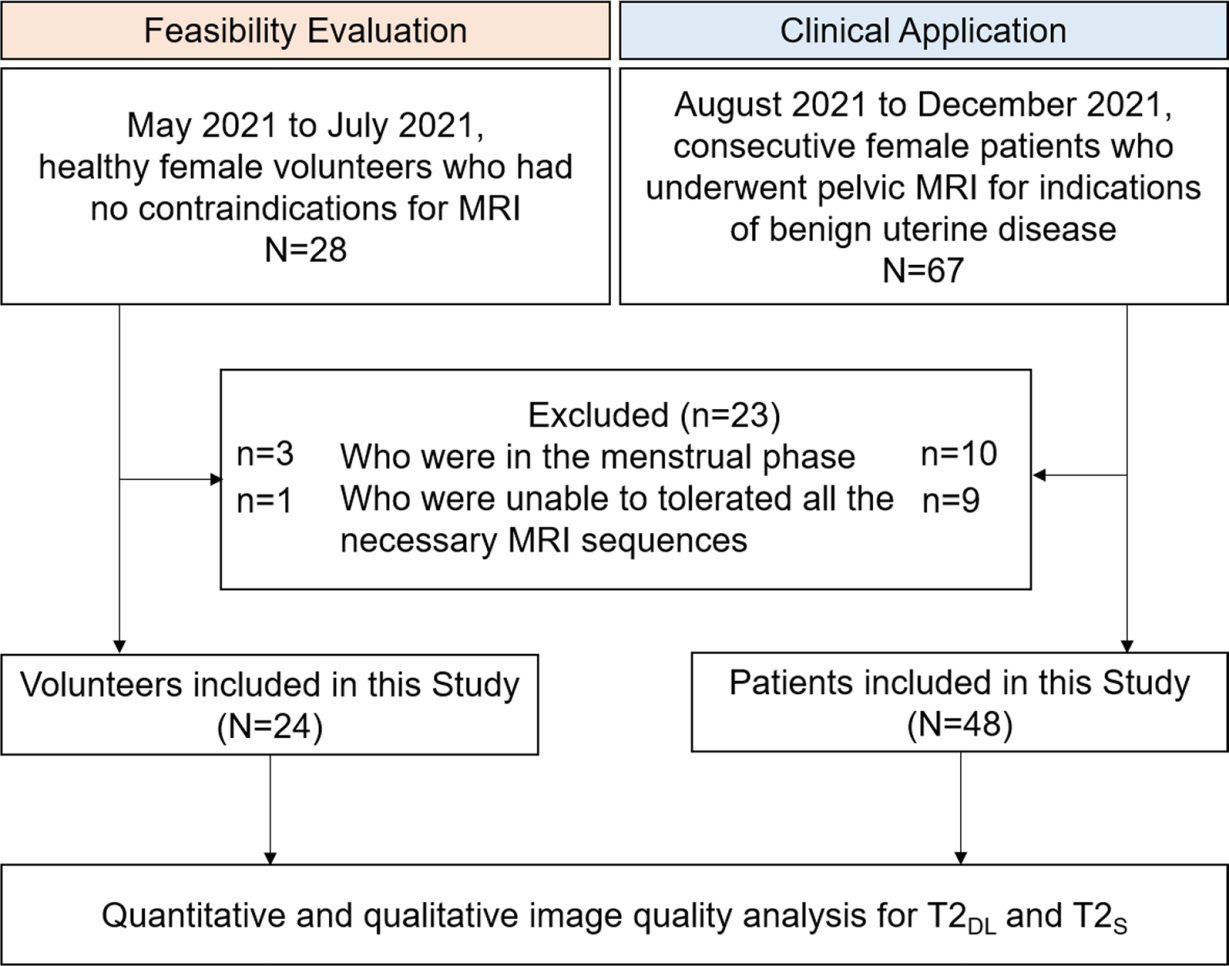
Flowchart of volunteer and patient inclusion process. T2_DL_, deep learning**-**accelerated T2-weighted turbo spin echo sequence; T2_S_, standard T2-weighted turbo spin echo sequence

This prospective study was approved by our institutional review board and complied with ethical committee standards. The sample size was estimated by considering both the difference in quantitative parameter means (using paired t-test/Wilcoxon signed-rank test) or qualitative evaluation scores (using Wilcoxon signed-rank test) between the DL-accelerated turbo spin echo (TSE) sequence (T2_DL_) and standard T2-weighted TSE sequence (T2_S_). Sample size estimation was conducted using G*Power 3 (version 3.1.9.7), and the statistical test was set as “matched pairs—different between two dependent means/Wilcoxon signed-rank test”. The error was set at 0.05, and the power level was set to 80–95%. Therefore, a total sample size of 22–38 was estimated when considering the difference in quantitative parameter means between T2_DL_ and T2_S_, while a sample size of 23–39 was obtained when considering the difference in qualitative evaluation score between T2_DL_ and T2_S_. The latter was selected as the target sample size for the study because it was larger. Then, participants in the study were enrolled in two stages: volunteers enrolled for feasibility evaluation and patients enrolled for clinical application (Fig. 1). Considering that T2_DL_ has not been used in female pelvic MRI before, we firstly recruited 24 healthy female volunteers who had no contraindications for MRI between May 2021 and July 2021 to preliminarily explore the feasibility of T2_DL_. Then, between August 2021 and December 2021, a totally of 48 consecutive female patients who underwent pelvic MRI for indications of benign uterine disease were enrolled (Table 1). Participants in the menstrual phase were exclude as boundary sharpness of the uterine zonal layers is reduced in this phase [21]. Written informed consent was obtained from participants.

**Table 1:** Characteristics of the study population

Characteristics	
Total (*n*. %)	72 (100)
Age (years)	35.50 (25.25–42.75)
BMI (kg/cm^2^)	21.58 (19.73–23.81)
Volunteers (*n*. %)	24 (33)
Age (year)	25.00 (24.00–26.75)
BMI (kg/cm^2^)	19.73 (18.62–21.51)
Patients (*n*. %)	48 (67)
Age (year)*	39.81 ± 8.95
BMI (kg/cm^2^)	22.77 (21.25–24.15)
Disease (*n*. %)	
Leiomyoma	27 (56)
Adenomyosis	9 (19)
Scarred uterus	12 (25)

*BMI*, body-mass index

*Continuous variables conforming to a normal distribution are presented as means ± standard deviations- otherwise as median (interquartile range [IQR])

### MRI technique

Participants received a 10-mL glycerin enema in the rectum 30 min before MRI to reduce air in the rectum and sigmoid. A moderately full bladder was required [5]. MRI studies were performed using a 3 T MR system (MAGNETOM Vida, Siemens Healthcare, Erlangen, Germany) with an 18-channel body phased-array receive coil. Unenhanced female pelvic MR protocol was performed using the following sequences: standard T2-weighted TSE sequence (T2_S_) in axial, sagittal, and coronal planes; T2WI acquired with a prototypic DL-accelerated TSE sequence (T2_DL_) (Siemens Healthcare, Erlangen, Germany) in axial, sagittal, and coronal planes; axial T1-weighted imaging (T1WI); and DWI with two *b* values in the axial plane (50 and 800 s/mm^2^). T2_DL_ was acquired after T2s to ensure the standard-of-care practice for clinical MRI. The detailed parameters for both T2W protocols are listed in Table 2.

**Table 2:** Detail T2-weighted MR imaging parameters for T2_DL_ and T2_S_ sequences

	Sagittal / Axial / Coronal	
	T2_DL_	T2_S_
Repetition time (ms)	3000	3000
Echo time (ms)	95	95
Field of view (mm^2^)	200 × 200	200 × 200
Matrix	432 × 367	432 × 367
Slice thickness (mm)	4	4
No. of slices	28	28
Reconstructed voxel size (mm^3^)	0.2 × 0.2 × 4.0	0.2 × 0.2 × 4.0
Parallel acceleration factor	3	3
Averages	1	2
Acquisition time (min:sec)	1:54	5:06

*T2*_*DL*_ deep learning-accelerated T2-weighted turbo spin echo sequence; *T2*_*S*_ standard T2-weighted turbo spin echo sequence

### Quantitative image evaluation

All axial, sagittal, and coronal T2W images (T2_S_ and T2_DL_) were randomized, anonymized, and independently evaluated using image-viewing software (RadiAnt DICOM Viewer 2020.1). Quantitative image evaluation was performed by a radiologist with 3 years of experience interpreting pelvic MR images. Whenever possible, operator-defined ROIs were placed on sagittal T2w images in the myometrium, junctional zone, gluteal muscle, and leiomyoma. The ROIs were as large as possible and were devoid of severe artifacts. The ROIs in the gluteal muscle were relatively small because care was taken to avoid areas of macroscopic fat.

The SD of the background signal intensity (SI) of images was measured as the noise value. The SNR for the myometrium, junctional zone, and leiomyoma is calculated according to Eq. 1 [22, 23]:

1 $${\text{SNR}} = \frac{{{\text{SI}}}}{{\text{N}}}$$

where SI is the mean SI within the ROIs, and N is the background noise. The CNR was then determined between the (1) myometrium and junctional zone, (2) myometrium and gluteal muscle, (3) junctional zone and gluteal muscle, and (4) myometrium and leiomyoma, according to Eq. 2 [22; 23]:

2 $${\text{CNR}} = \frac{{{\text{SI}}_{{\text{A}}} - {\text{SI}}_{{\text{B}}} }}{{\text{N}}},$$

where SI_A_ and SI_B_ are the mean SIs of the two tissue types mentioned previously. Care was taken to size and place the ROIs consistently for each pair of images from the same patient. In patients with multiple lesions, only the largest lesions were analyzed.

Geometric image distortion was evaluated by measuring the diameters in all three directions of the uterus and lesions depicted on both T2_DL_ and T2_S_ images. For the 24 healthy volunteers, the long (i.e., parallel to the long axis of the uterine body), short (i.e., maximum length perpendicular to the long diameter), and transverse (i.e., distance between the two uterine horns) diameters of the uterus were measured on both T2_DL_ and T2_S_ slices in which the uterus appeared largest. Given the relative ease and accuracy of uterine leiomyoma as compared to adenomyosis and scarred uterus measurements, for each affected patient (n = 27), T2_DL_ and T2_S_ slices with the maximum lesion area were examined for the largest lesion, followed by measurements of the anteroposterior, vertical, and transverse diameters of this lesion.

### Qualitative image evaluation

All anonymized image datasets were independently evaluated in a random order by three radiologists with 3, 5, and 11 years of experience in interpreting pelvic MR images. The readers were blinded to the sequence types, as image information was concealed in the viewing software. Overall image quality, artifacts, boundary sharpness of the uterine zonal layers, and lesion conspicuity on axial, coronal, and sagittal images were rated by the radiologists using a 5-point Likert scale, with 1 and 5 representing the poorest and best performance, respectively (Table 3). In patients with multiple lesions, only a single score was assigned after the consideration of all lesions.

**Table 3:** Qualitative image evaluations based on the 5-point Likert scale

Score	Overall image quality	Artifacts	Boundary sharpness of the uterine zonal layers	Lesion conspicuity
1	Non-diagnostic	Non-diagnostic	Unable to see	Lesion unidentifiable
2	Substantial deficits in image quality	Substantial impact on diagnosis	Blurry but visualized	No differentiation between lesion and normal anatomy
3	Moderate image quality	Moderate impact on diagnosis	Acceptable	Subtle lesion with poorly defined edges
4	Good image quality	Little impact on image diagnosis	Good	Well-seen lesion with poorly defined edges
5	Excellent image quality	No artifact	Excellent	Well-seen lesion with well-defined edges

### Statistical analysis

Statistical analysis was performed using SPSS version 21.0.0.0 (IBM; https://www.ibm.com). After testing for normality using the Kolmogorov–Smirnov test, continuous variables conforming to a normal distribution are presented as means ± standard deviations, otherwise as median (interquartile range [IQR]). Ordinal scaled variables are presented as median (IQR). The results of quantitative analysis were compared using a paired *t*-test for normally distributed variables, or Wilcoxon signed-rank test for nonnormally distributed variables. The distribution and concordance of uterine and lesion diameters in all three directions were obtained on both T2_DL_ and T2_S_ images and were presented in Bland–Altman plots. Qualitative scores were compared using the Wilcoxon signed-rank test. Interobserver agreement was evaluated using the Kendall W test (≤ 0.4, poor agreement; 0.41–0.75, good agreement; > 0.75, excellent agreement). Two-tailed *p* values < 0.05 were considered statistically significant.

## Results

### Patient characteristics

In total, 72 participants (median [IQR] age: 35.50 [25.25–42.75] years; range: 23–71 years) were included in this study. The body mass index of participants was 21.58 (19.73–23.81) kg/cm^2^ (range: 16.94–37.20 kg/m^2^). Among the 48 patients with benign pelvic diseases, 27 had uterine leiomyoma, 9 had adenomyosis, and 12 had a scarred uterus.

### Quantitative image evaluation

**Fig. 2:**
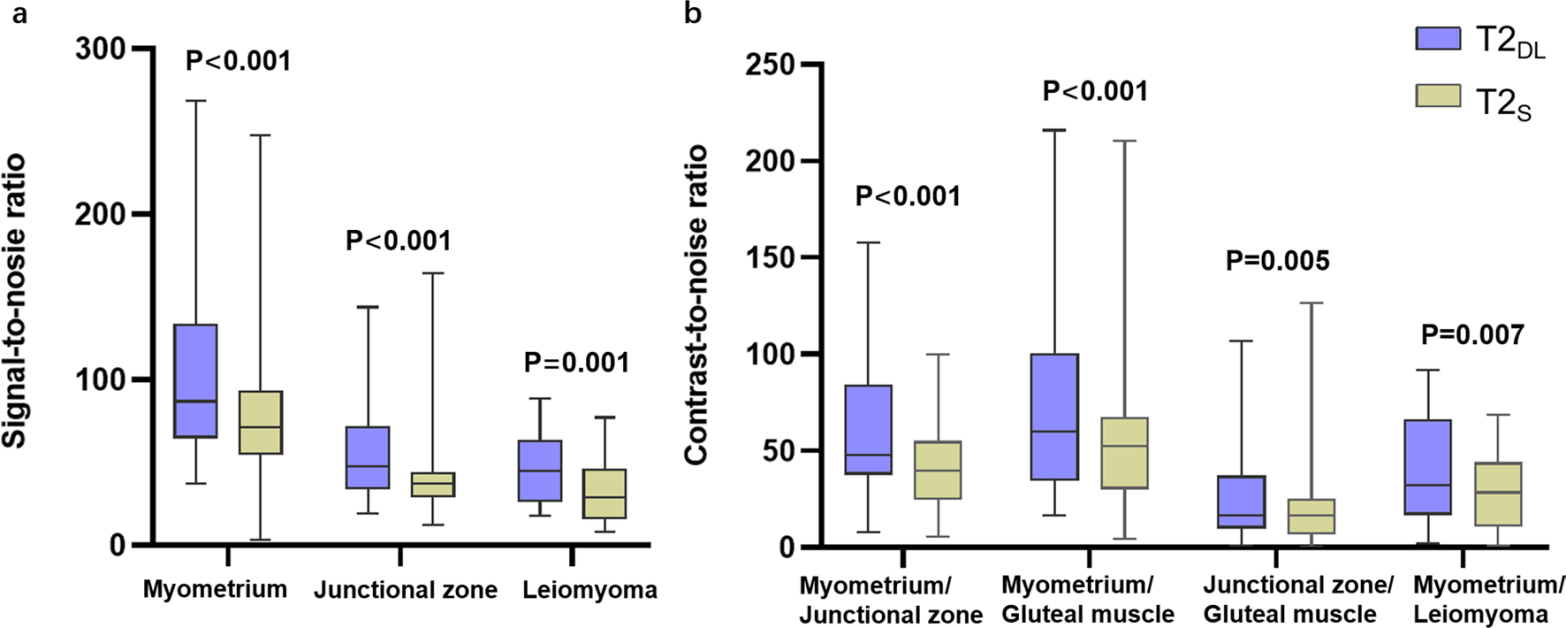
Box and whisker plots showing the results for SNRs and CNRs using T2w images acquired with T2_DL_ and T2_S_ during MRI of the female pelvis. **a** SNRs of the myometrium, junctional zone, and leiomyoma were significantly higher for T2_DL_ than for T2_S_ (*p* < *0.001*, *p* < *0.001*, and *p* = *0.001*, respectively). **b** CNRs between the myometrium and junctional zone, between the myometrium and gluteal muscle, between the junctional zone and gluteal muscle, and between the myometrium and leiomyoma were significantly higher for T2_DL_ than for T2_S_ (*p* < *0.001*, *p* < *0.001*, *p* = *0.005*, and *p* = *0.007*, respectively). Center line = median; top of box = 75th percentile; bottom of box = 25th percentile; whiskers = smallest and largest nonoutlier values. *SNR* signal-to-noise ratio; *CNR* contrast-to-noise ratio; *T2*_*DL*_ deep learning**-**accelerated T2-weighted turbo spin echo sequence; *T2*_*S*_ standard T2-weighted turbo spin echo sequence

The acquisition time of axial, sagittal, and coronal imaging was 1:54 min and 5:06 min for T2_DL_ and T2_S_, respectively. The total acquisition time, including all T2-weighted sequences, was 5:42 min and 15:18 min for T2_DL_ and T2_S_, respectively. Overall, the acquisition time of T2_DL_ images was reduced by 62.7% compared to that of T2_S_ images. A total of 432 image datasets were independently evaluated. The median (IQR) or mean ± SD areas of ROIs in the myometrium, junctional zone, gluteal muscle, leiomyoma, and image background were 69.10 (52.83–76.93) mm^2^, 11.30 (8.10–17.65) mm^2^, 25.51 ± 10.69 mm^2^, 579.75 (153.70–1286.73) mm^2^, and 1052.03 ± 586.69 mm^2^, respectively. The mean SNRs of the myometrium, junctional zone, and leiomyoma were significantly higher on T2_DL_ images than on T2_S_ images (108.63 vs. 76.36, *p* < 0.001; 57.64 vs. 40.77, *p* < 0.001; and 44.98 vs. 32.86, *p* = 0.001, respectively) (Fig. 2a). The mean CNRs were also significantly higher on T2_DL_ images than on T2_S_ images: between the myometrium and junctional zone, 60.14 vs. 42.32, *p* < 0.001; between the myometrium and gluteal muscle, 76.43 vs. 54.73, *p* < 0.001; between the junctional zone and gluteal muscle, 25.63 vs. 19.47, *p* = 0.005; and between the myometrium and leiomyoma, 39.13 vs. 28.29, *p* = 0.007 (Fig. 2b).

**Fig. 3:**
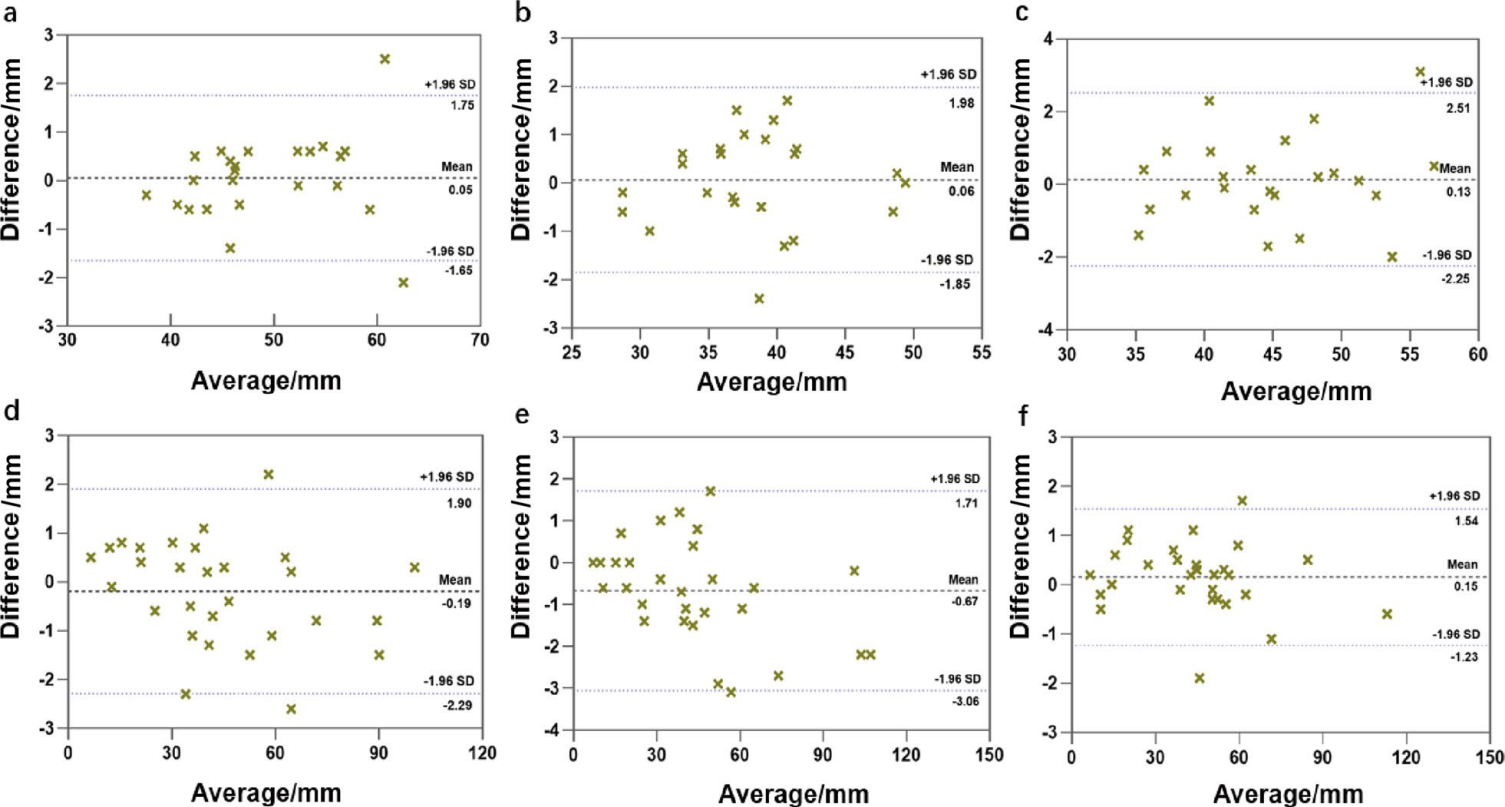
Bland–Altman plots showing differences versus averages in maximum diameters of the uterus and lesions in T2_DL_ and T2_S_ images of the female pelvis. **a**–**c** Measurements of the long (**a**), short (**b**), and transverse (**c**) diameters of the uterus. **d**–**f** Measurements of the anteroposterior (**d**), vertical (**e**), and transverse (**f**) diameters of leiomyoma lesions. Of the 24 uterine samples, only 2 (8.3%) long diameters, 1 (4.2%) short diameter, and 1 (4.2%) transverse diameter exceeded the 95% limits of agreement. Of the 29 leiomyoma lesion samples, 3 (10.3%) anteroposterior diameters, 2 (6.9%) vertical diameters, and 2 (6.9%) transverse diameters exceeded the 95% limits of agreement, indicating minor geometric distortion of T2_DL_ images. *T2*_*DL*_ deep learning**-**accelerated T2-weighted turbo spin echo sequence; *T2*_*S*_ standard T2-weighted turbo spin echo sequence

Quantitative measurements revealed no significant differences in maximum diameter of the uterus and leiomyoma lesions between the two sequences. Mean ± SD long, short, and transverse diameters of the uterine body were 49.29 ± 7.03 mm, 38.27 ± 5.58 mm, and 44.92 ± 6.39 mm, respectively, for T2_DL_ and 49.23 ± 6.89 mm, 38.20 ± 5.54 mm, and 44.80 ± 6.23 mm, respectively, for T2_S_ (*p* = 0.979, *p* = 0.969, and *p* = 0.944, respectively). The mean ± SD anteroposterior, vertical, and transverse diameters of the leiomyoma lesions were 44.22 ± 23.87 mm, 43.37 ± 26.65 mm, and 44.28 ± 23.51 mm, respectively, for T2_DL_ and 44.41 ± 24.17 mm, 44.04 ± 27.17 mm, and 44.12 ± 23.63 mm, respectively, for T2_S_ (*p* = 0.976, *p* = 0.925, and *p* = 0.981, respectively). Bland–Altman plots revealed minimal geometric distortion in the T2_DL_ images. The mean difference in percentage and limits of agreement between T2_DL_ and T2_S_ was − 0.67% to 0.15% (Fig. 3).

### Qualitative image evaluation

Interobserver agreement on qualitative image evaluation among the three readers was excellent (Kendall W coefficient: 0.801–0.903 for T2_DL_ and 0.771–0.914 for T2_S_; all *p* < 0.001). Images from 72 participants were used to evaluate the overall image quality, artifacts, and boundary sharpness of the uterine zonal layers and images from 37 patients with visible lesions were used to evaluate lesion conspicuity. The overall quality of sagittal, axial, and coronal images was rated higher for T2_DL_ (median [IQR] of 5 (4–5), 5 (4–5), and 5 (4–5), respectively) than for T2_S_ (median [IQR] of 4 (4–5), 4 (4–4), and 4 (4–4); all *p* < 0.001).

**Fig. 4:**
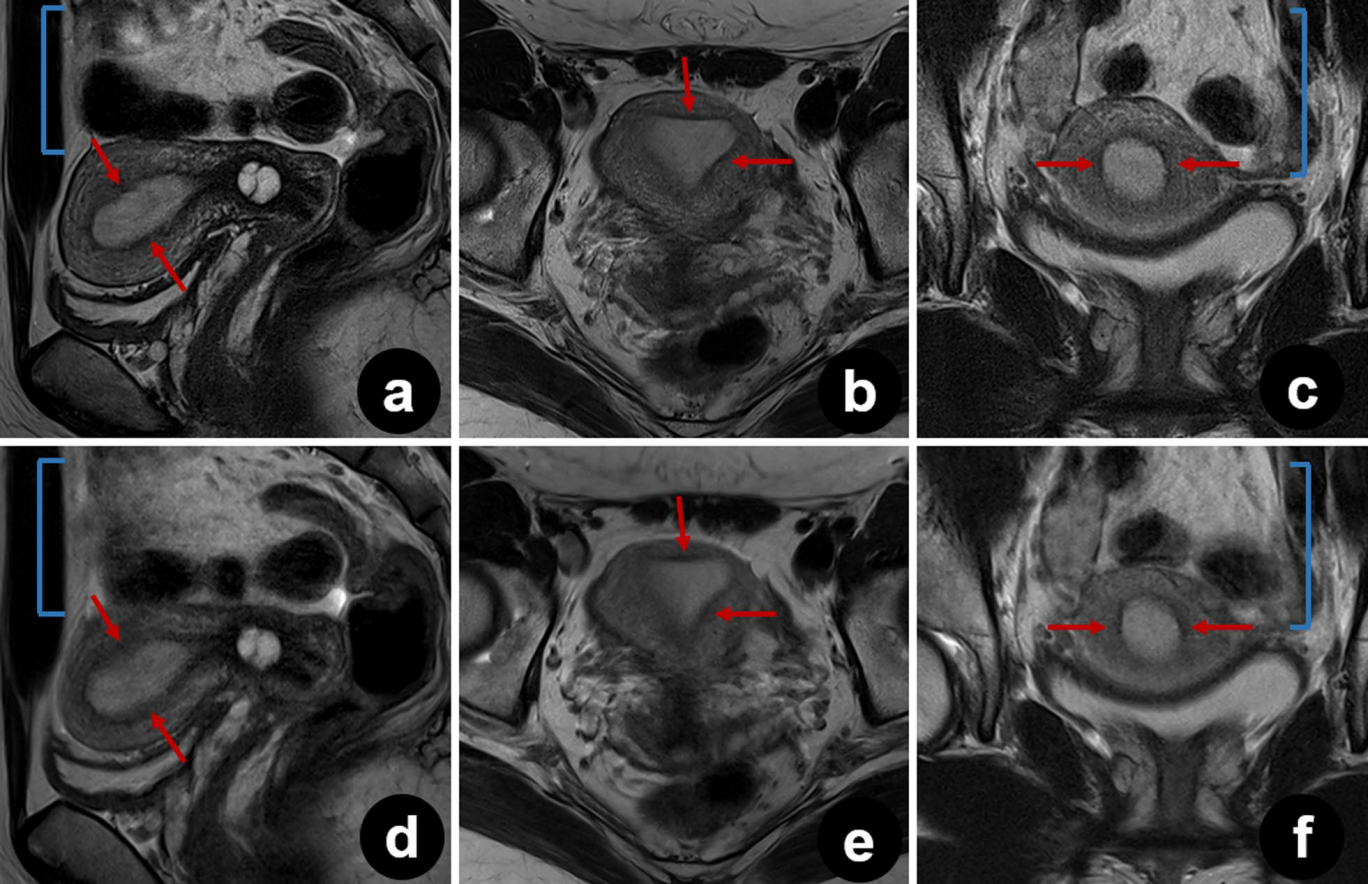
Pelvic T2_DL_ and T2_S_ images of a 25-year-old healthy female volunteer. **a**–**f** Sagittal (**a**), axial (**b**), and coronal (**c**) T2_DL_ images and sagittal (**d**), axial (**e**), and coronal (**f**) T2_S_ images. Fewer bowel peristalsis artifacts (brackets) were observed on T2_DL_ images, which show sharper depiction of the uterine zonal layers (arrows). Due to severe motion artifacts (brackets), three uterine zonal layers were not clearly depicted on T2_S_ images (arrows). T2_DL_, deep learning**-**accelerated T2-weighted turbo spin echo sequence; T2_S_, standard T2-weighted turbo spin echo sequence

**Fig. 5:**
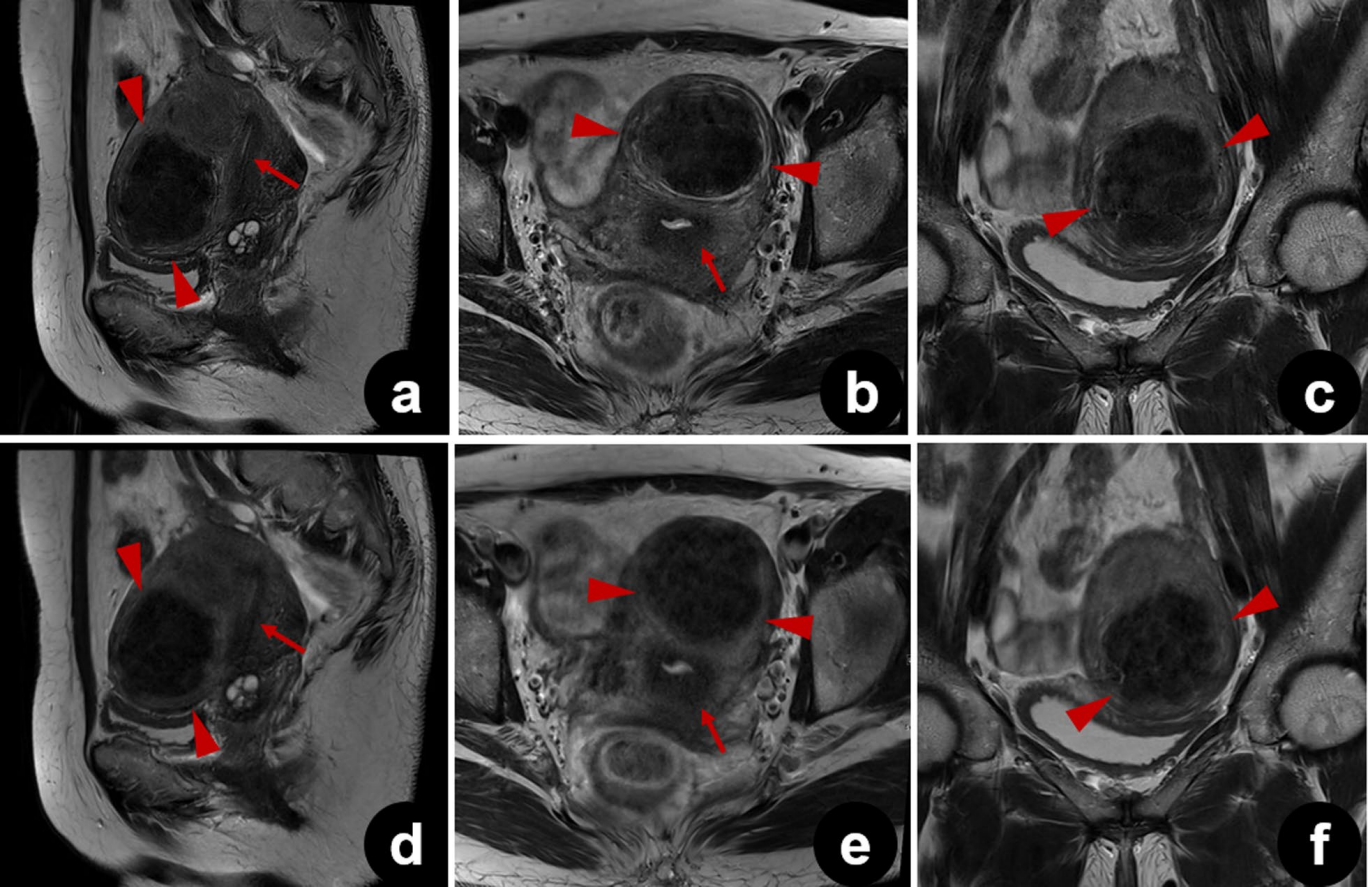
Pelvic T2_DL_ and T2_S_ images of a 47-year-old female patient with leiomyoma. **a**–**f** Sagittal (**a**), axial (**b**), and coronal (**c**) T2_DL_ images and sagittal (**d**), axial (**e**), and coronal (**f**) T2_S_ images. The edge and internal structure of the tumor were most clearly delineated by T2_DL_ (between arrowheads). Note that sharper uterine zonal layers are also observed on T2_DL_ images (arrows). *T2*_*DL*_ deep learning**-**accelerated T2-weighted turbo spin echo sequence; *T2*_*S*_ standard T2-weighted turbo spin echo sequence

**Fig. 6:**
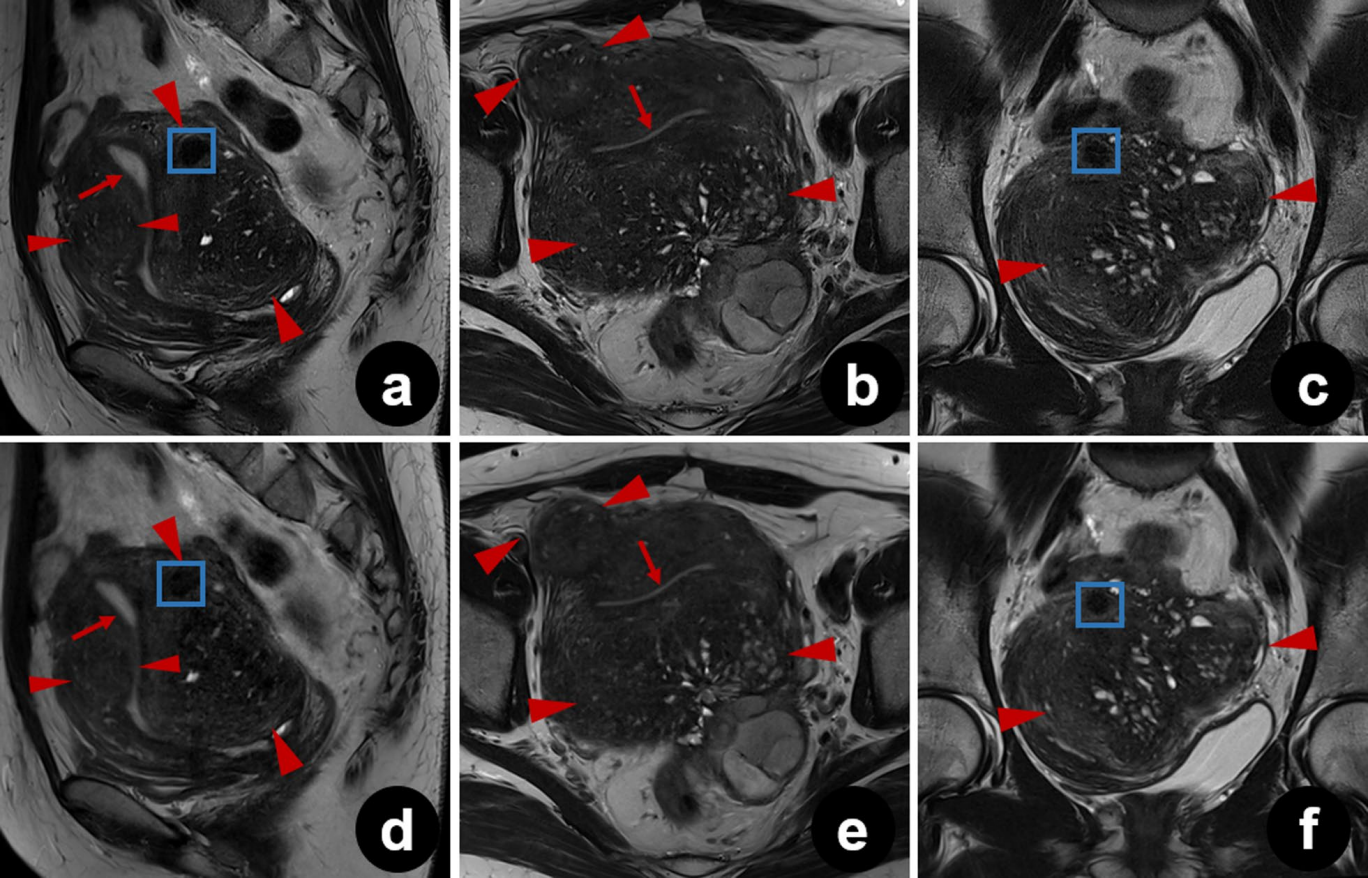
Pelvic T2_DL_ and T2_S_ images of a 43-year-old female patient with diffuse adenomyosis. Sagittal (**a**), axial (**b**), and coronal (**c**) T2_DL_ images and sagittal (**d**), axial (**e**), and coronal (**f**) T2_S_ images. Typical findings of asymmetry of uterine wall thickness, poorly demarcated low-SI area, and intramyometrial cysts are presented. Compared to T2_S_ images, T2_DL_ images exhibited greater conspicuity of the lesions (between arrowheads) and uterine zonal layers (arrows). Note that a small leiomyoma is also observed more clearly on sagittal and coronal T2_DL_ images (frame) than on the corresponding T2_S_ images. *SI* signal intensity; *T2*_*DL*_ deep learning**-**accelerated T2-weighted turbo spin echo sequence; *T2*_*S*_ standard T2-weighted turbo spin echo sequence

Ghost artifacts were detected in T2_S_ images from all patients. Artifacts were also observed in T2_DL_ images, but they were significantly reduced in some sagittal, axial, and coronal images (median [IQR] scores for T2_S_ and T2_DL_ were 4 [4–5] and 5 [4–5], 4 [4–5] and 5 [5–5], and 4 [4–5] and 5 [4–5], respectively, all *p* < 0.001) (Figs. 4, 5, and 6). In addition to a reduced amount of motion artifacts, T2_DL_ images exhibited significantly higher conspicuity of the uterine zonal layers and lesions (Figs. 5 and 6). Scores for boundary sharpness of the zonal layers were significantly higher for T2_DL_ than for T2_S_. Median (IQR) scores for T2_DL_ and T2_S_ were 5 (5–5) and 4 (4–5), 5 (4–5) and 4 (4–4), and 5 (4–5) and 4 (4–4), respectively in sagittal, axial, and coronal images (all *p* < 0.001). Furthermore, lesion conspicuity was rated superior for T2_DL_ compared with T2_S_, with a median (IQR) of 5 (4–5) versus 4 (4–4), respectively, for both sagittal and axial images and a median (IQR) of 5 (4–5) versus 4 (4–5) for coronal images (*p* = 0.002, *p* < 0.001, and *p* = 0.021, respectively). The results of quantitative image evaluation of T2_DL_ and T2_S_ images by the three readers are presented in Table 4.

**Table 4:** Qualitative image evaluations and inter-reader agreement for T2_DL_ and T2_S_ sequences

Characteristics	Reader 1			Reader 2			Reader 3			Kendall’s W	
	T2_DL_	T2_S_	*p* values	T2_DL_	T2_S_	*p* values	T2_DL_	T2_S_	*p* values	T2_DL_	T2_S_
*Sagittal*											
Overall image quality	5 (4–5)	4 (4–5)	< 0.001	5 (4–5)	4 (4–5)	< 0.001	5 (4–5)	4 (4–5)	< 0.001	0.873	0.887
Artifacts	5 (4–5)	4 (4–5)	< 0.001	5 (4–5)	4 (4–5)	< 0.001	5 (4–5)	4 (4–5)	< 0.001	0.903	0.914
Boundary sharpness	5 (4–5)	4 (4–5)	< 0.001	5 (4–5)	4 (4–5)	< 0.001	5 (5–5)	4 (4–5)	< 0.001	0.801	0.771
Lesion conspicuity	5 (5–5)	4 (4–4)	< 0.001	5 (4–5)	4 (4–4)	< 0.001	5 (4–5)	4 (4–4)	0.002	0.847	0.871
*Axial*											
Overall image quality	5 (4–5)	4 (4–4)	< 0.001	5 (4–5)	4 (4–4)	< 0.001	5 (4–5)	4 (4–4)	< 0.001	0.851	0.871
Artifacts	5 (4–5)	4 (4–4)	< 0.001	5 (4–5)	4 (4–4)	< 0.001	5 (5–5)	4 (4–5)	< 0.001	0.840	0.828
Boundary sharpness	5 (4–5)	4 (4–4)	< 0.001	5 (4–5)	4 (4–4)	< 0.001	5 (4–5)	4 (4–4)	< 0.001	0.828	0.841
Lesion conspicuity	5 (4–5)	4 (4–4)	< 0.001	5 (5–5)	4 (4–4)	< 0.001	5 (4–5)	4 (4–4)	< 0.001	0.896	0.884
*Coronal*											
Overall image quality	5 (4–5)	4 (4–4)	< 0.001	5 (4–5)	4 (4–4)	< 0.001	5 (4–5)	4 (4–4)	< 0.001	0.867	0.905
Artifacts	5 (4–5)	4 (4–5)	< 0.001	5 (4–5)	4 (4–4)	< 0.001	5 (4–5)	4 (4–5)	< 0.001	0.876	0.890
Boundary sharpness	5 (4–5)	4 (4–4)	< 0.001	5 (4–5)	4 (4–4)	< 0.001	5 (4–5)	4 (4–4)	< 0.001	0.903	0.882
Lesion conspicuity	5 (4–5)	4 (4–4)	0.001	5 (4–5)	4 (4–4)	0.003	5 (4–5)	4 (4–5)	0.021	0.864	0.879

*T2*_*DL*_ deep learning-accelerated T2-weighted turbo spin echo sequence; *T2*_*S*_ standard T2-weighted turbo spin echo sequence

## Discussion

This prospective study first compared the feasibility of a T2_DL_ with that of a T2_S_ for gynecological MRI in healthy volunteers and patients with benign uterine disease. Compared to T2_S_, T2_DL_ produced images with markedly improved SNR and CNR without geometric distortion. Further, T2_DL_ reduced the acquisition time by 62.7%. In addition, T2_DL_ images displayed superior image quality, boundary sharpness of the uterine zonal layers, and lesion conspicuity of benign uterine disease, as well as fewer artifacts in the three planes.

In previous studies of the DL reconstruction technique for accelerated abdominal and prostate MR acquisitions, noise was subjectively rated as superior to that of standard sequence images [20, 24]. In the present study, the DL-accelerated technique was embedded in the female pelvic MRI protocol to acquire images for the first time. We designed to calculate the SNR an CNR values using the most commonly used measurement method (separate signal and noise regions in a single image), to objectively compare the image quality of T2_DL_ and T2_S_. Some previously published studies revealed that such measurement method may degrade the SNR performance in parallel imaging, as noise is not evenly distributed in accelerated images reconstructed with parallel imaging [25, 26]. However, we aimed to compare the SNR and CNR values in two sequences (T2_DL_ vs. T2_S_) both with parallel imaging reconstruction, and the acquisition parameters, including repetition time, echo time, echo train length, and parallel acceleration factor used in T2_DL_ are identical to those used in T2_S_. The ROIs on the image background were placed as large as possible (mean area: 1052.03 mm^2^) and were devoid of artifacts to reduce the impact of noise heterogeneity on the quantitative analysis. We found that although the mean SIs within the ROIs of the two sequences were similar in this study, T2_DL_ images demonstrated considerably lower background noise intensity compared to T2_S_ images. Therefore, the TSE sequence with DL reconstruction resulted in higher SNR and CNR values relative to the conventional approach. In addition, a higher CNR between the myometrium and junctional zone may have enhanced the boundary sharpness of the uterine zonal layers, as T2_DL_ images were rated superior to T2_S_ images on boundary sharpness evaluation by all three readers.

Motion artifacts pose a major challenge in pelvic MRI. Periodic motion from the lower anterior abdominal wall and pulsatile vessels, as well as the random motion of the peristaltic viscera are key sources of artifact generation [27, 28]. The use of rapid image sequences may directly minimize random motion and facilitate breath-holding, thereby reducing motion artifacts [10, 11, 27]. Tsuboyama et al. reported that periodic motion produced marked ghost artifacts on conventional TSE, but not on half-Fourier acquisition single-shot turbo spin echo (HASTE) sequences, because the acquisition time was significantly shorter with HASTE than with TSE [29]. As shown in Fig. 4, bowel peristalsis and ghost artifacts were significantly reduced in T2_DL_ images. Consequently, the effect of motion artifacts was smaller for T2_DL_ than for T2_S_, resulting in more clearly delineated lesion edges and internal structures. Typical findings of adenomyosis, including poorly demarcated low-SI area, intramyometrial cysts, and small high-SI areas on T2W images, were more sharply depicted on T2_DL_ than on T2_S_, as shown in Fig. 6 [30]. The finding that motion-robust T2_DL_ images were evaluated as superior to T2_S_ images suggests that these accelerated images may be clinically acceptable.

Gynecological MRI has been recognized as the optimal imaging modality for the assessment of pelvic diseases in women. Accordingly, the DL reconstruction technique, which accelerates gynecological imaging, offers relevant clinical benefits [2, 29, 31]. The shorter sequencing time may reduce MRI costs and permit greater patient throughput and/or more imaging to be completed per unit time. Furthermore, fast imaging sequences may relieve patient anxiety, thereby allowing the acquisition of motion-robust images with optimal diagnostic value. In future, the accelerated gynecological MRI protocol may be harnessed as a candidate or even a replacement for conventional protocols.

This study has several limitations. First, as the study involved preliminary experiments using a DL reconstruction technique for gynecological MRI, the study population was small and was derived from a single center. Future multicenter studies involving larger cohorts are needed to evaluate the robustness and diagnostic ability of T2_DL_ images. Second, since varying noise distributions resulting from the deployed neural networks may exist, the validity of the SNR and CNR measurement in the present study is not clear. Measurement methods applicable for MR images with the DL reconstruction should be proposed for quantitative evaluation in future studies. Third, patients we included had different benign lesion types, which could potentially affect subjective and objective analyses. Future studies involving patients with the same lesion type are recommended. Finally, the conclusions may have limited generalizability, because only healthy volunteers and patients with benign uterine diseases were enrolled. The image quality and diagnostic performance of T2_DL_ images in women with other pelvic diseases, especially gynecologic malignancies, remain to be elucidated. In this regard, the clinical value of this DL reconstruction technique should be further explored for other gynecological diseases.

In conclusion, the DL-accelerated T2w TSE sequence is an effective and promising approach that reduces the acquisition time of female pelvic MRI compared to the standard TSE sequence, with the added benefits of significantly improved image quality, boundary sharpness, and lesion conspicuity in patients with benign uterine disease.

## Data Availability

The datasets analyzed during the current study are available from the corresponding author on reasonable request.

## Abbreviations

CNR: Contrast-to-noise ratio
CS: Compressed sensing
DL: Deep learning
IQR: Interquartile range
SD: Standard deviation
SI: Signal intensity
SNR: Signal-to-noise ratio
T2_DL_: Deep learning**-**accelerated T2-weighted turbo spin echo sequence
T2_S_: Standard T2-weighted turbo spin echo sequence
T2WI: T2-weighted imaging
TSE: Turbo spin echo
